# Anti-Inflammatory Activity of Velvet Bean (*Mucuna pruriens*) Substances in LPS−Stimulated RAW 264.7 Macrophages

**DOI:** 10.3390/molecules27248797

**Published:** 2022-12-12

**Authors:** Dong-Geun Han, Min-Jun Bae, Bong-Jeon An

**Affiliations:** Department of Cosmeceutical Science, Daegu Hanny University, Gyeongsan 712715, Republic of Korea

**Keywords:** *Mucuna pruriens*, macrophage, lipopolysaccharide, anti-inflammatory, L-DOPA

## Abstract

In this study, *Mucuna pruriens* extracts were used to verify their application as a natural-based raw material with anti-inflammatory function. A nitric oxide inhibition activity assay showed that *M. pruriens* extracted with hot water (MW), *M. pruriens* extracted with 70% ethanol (ME), and *M. pruriens* extracted with 70% acetone (MA) presented NO inhibition activity; among them, MW and ME demonstrated the best activity and were selected for Western blot analysis. After identifying the expression patterns of inflammation-related proteins, such as inducible nitric oxide synthase (iNOS), cyclooxygenase-2 (COX-2), c-jun N-terminal kinase (JNK), extracellular signal-regulated kinase (ERK), and nuclear factor kappa-light-chain-enhancer of activated B cell (NF-κB), through Western blots, both MW and ME showed inhibition patterns. As a result of analyzing L-DOPA contained in *M. pruriens* through ultra-performance liquid chromatography (UPLC), high L-DOPA content was detected in MW, ME, and MA. Therefore, it can be concluded that *M. pruriens* extracts have the potential for use as an anti-inflammatory material.

## 1. Introduction

*Mucuna pruriens* is an annual leguminous plant that is widely distributed in tropical and subtropical regions of the world and is used as a cover crop for feed and green manure. *M. pruriens* is highly valued as a medicine for various diseases such as sexual dysfunction, Parkinson’s disease, and brain-related diseases, among others [1,2]. *M. pruriens* contains the highest levels of L-3,4-dihydroxyphenylalanine (L-DOPA) among all plants; L-DOPA is well-known for alleviating progressive disorders and Parkinson’s disease associated with dopamine deficiency [3]. In addition, *M. pruriens* contains ingredients such as alkaloids, amino acids, fatty acids, lecithin, tryptamine, tannin, and polyphenols, and has various pharmacological uses, e.g., as an aphrodisiac, antidiabetic, antioxidant, and anti-inflammatory [4].

When inflammation occurs, nitric oxide (NO) works as a signaling molecule that induces vasodilation and the immune response of macrophages; excessive generation of NO develops into chronic inflammation with the continued inflammatory response, leading to allergic diseases, asthma, and arthritis, among other issues [5,6]. iNOS is expressed in response to lipopolysaccharide (LPS), interferon-gamma (IFN-γ), etc.; biosynthesizes NO; and is known to be involved in causing skin erythema, dermatosis, and an inflammatory response, as well as in regulating immune function [7]. In addition, the expressed iNOS activates COX−2, and the activated COX−2 is involved in the production of prostaglandin E_2_ (PGE_2_), an inflammatory reaction mediator; this PGE_2_ is known to act on inflammatory diseases, autoimmune diseases, pain, and fever, among other conditions [8]. Therefore, we also focused on iNOS, COX−2, JNK, ERK1/2, and NF-κB, which are relatively well-known inflammation-related gene expressions.

Through the verification of pharmacological anti-inflammatory activity, in this study, we aimed to identify *M. pruriens* extracts’ applicability as a natural functional material.

## 2. Results and Discussion

### 2.1. Cell Viability

The 3-4,5-dimethylthiazol-2-yl-2,5-diphenyltetrazolium bromide (MTT) assay has various advantages: it provides quantifiable, objective results; it is relatively simple to perform; and it only requires a duration of 3–5 days to carry out the experiment [9].

**Figure 1:**
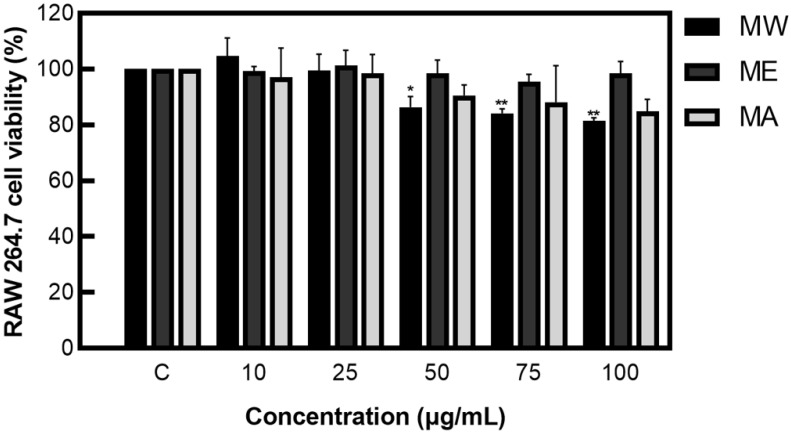
Cell viability of *M. pruriens* extracts on macrophage cell (RAW 264.7). MW: *M. pruriens* extracted with hot water; ME: *M. pruriens* extracted with 70% ethanol; MA: *M. pruriens* extracted with 70% acetone. Result are means ± SD of triplicate data (significantly different according to the Tukey test and significant compared to the control. * *p* < 0.05, ** *p* < 0.01).

After measuring cell viability, a concentration of 100 µg/mL or less, which showed a viability of 80% or more, was applied to the cell experiments, preventing the issue of cell population degradation due to cytotoxicity or denaturation (Figure 1).

### 2.2. Nitric Oxide (NO) Inhibition Activity of Mucuna Pruriens Extracts

**Figure 2:**
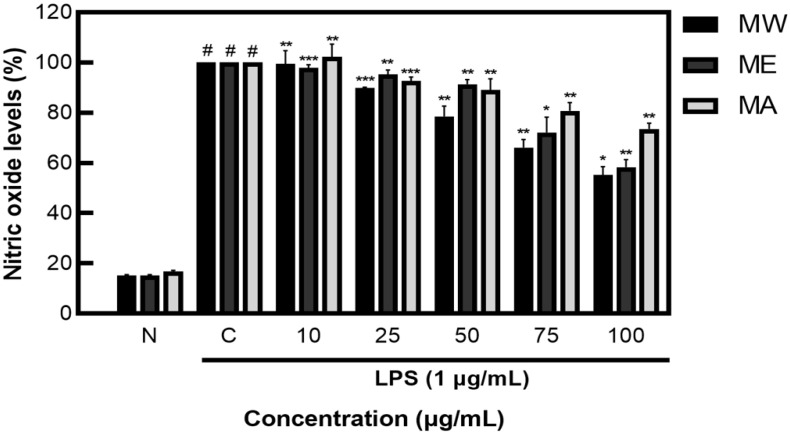
Inhibition rate of nitric oxide (NO) by *M. pruriens* extracts in macrophage cell (RAW 264.7). MW: *M. pruriens* extracted with hot water; ME: *M. pruriens* extracted with 70% ethanol; MA: *M. pruriens* extracted with 70% acetone. N: normal, not treated with LPS, C: control, LPS-treated. Results are means ± SD of triplicate data (significantly different according to a Tukey test and significant as compared to control. # *p* < 0.05 vs. non-treated N, * *p* < 0.05, ** *p* < 0.01, *** *p* < 0.001).

After measuring NO inhibition activity on *M. pruriens* extracts, less than 20% NO was produced in the group not treated with LPS, showing NO inhibition activity of 44.80% for MW, 41.60% for ME, and 26.49% for MA (Figure 2). These results demonstrate that *M. pruriens* extracts decreased the production of NO in LPS-stimulated RAW 264.7 macrophages.

### 2.3. Anti-Inflammatory Activity of Mucuna Pruriens Extracts

**Figure 3:**
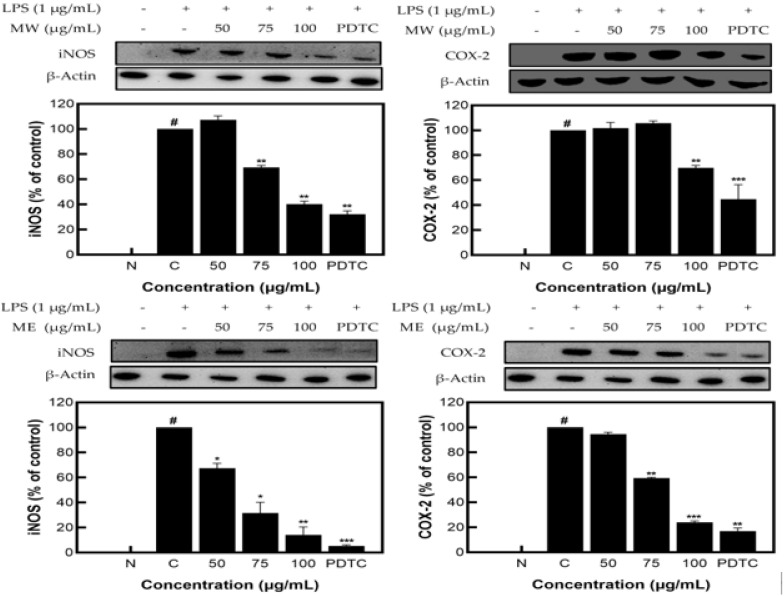
iNOS and COX−2 protein expression rate of *M. pruriens* extracts on macrophage cell (RAW 264.7). MW: *M. pruriens* extracted with hot water; ME: *M. pruriens* extracted with 70% ethanol; PDTC: ammonium pyrrolidine dithiocarbonate. N: normal, not treated with LPS; C: control, LPS-treated. Results are means ± SD of triplicate data (significantly different according to a Tukey test and significant as compared to control. # *p* < 0.05 vs. non-treated N, * *p* < 0.05, ** *p* < 0.01, *** *p* < 0.001).

MW, ME, and MA showed excellent NO inhibition activity; among them, MW and ME showed the best activity and were selected for Western blot analysis. The results identifying the expression patterns of iNOS and COX−2 proteins on *M. pruriens* extracts showed decreases in the expression of iNOS and COX−2 at 62.56% and 31.51% for MW and 78.73% and 75.05% for ME, respectively, at the highest concentration of 100 μg/mL. These results suggest that *M. pruriens* extracts decreased iNOS and COX−2 protein expression related to inflammation (Figure 3).

**Figure 4:**
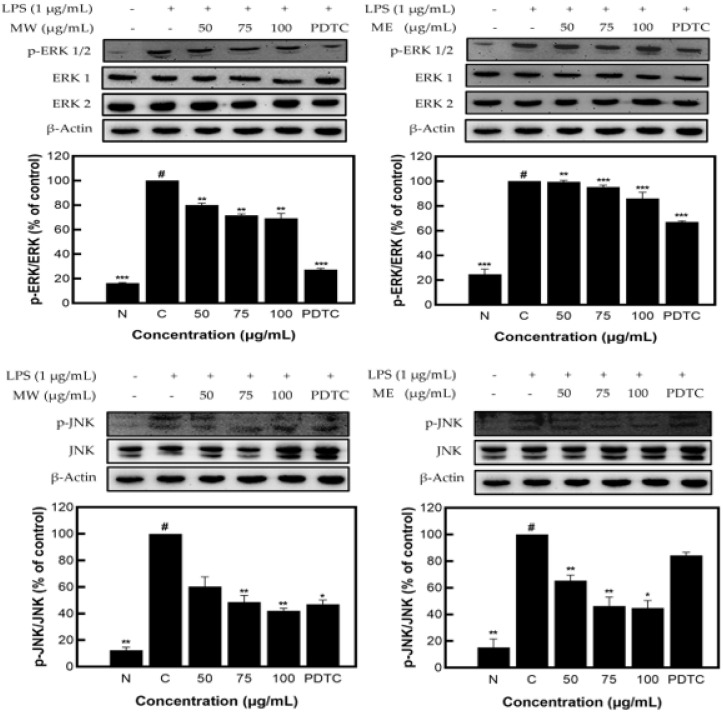
JNK, p-JNK and ERK1/2, p-ERK1/2 protein expression rate of *M. pruriens* extract on macrophage cell (RAW 264.7). MW: *M. pruriens* extracted with hot water; ME: *M. pruriens* extracted with 70% ethanol; PDTC: ammonium pyrrolidine dithiocarbonate. N: normal, not treated with LPS; C: control, LPS-treated. Results are means ± SD of triplicate data (significantly different according to a Tukey test and significant as compared to the control. # *p* < 0.05 vs. non-treated N, * *p* < 0.05, ** *p* < 0.01, *** *p* < 0.001).

Figure 4 shows the decreased expression patterns of p-JNK and p-ERK1/2 as compared to JNK and ERK1/2, presented through Western blots. Similar to the identified expression pattern results of the JNK, p-JNK, ERK1/2, and p-ERK1/2 proteins in *M. pruriens* extracts, MW showed a decrease in expression of JNK/p-JNK and ERK1/2/p-ERK1/2 of 57.90% and 30.77%, and for ME, of 55.12% and 13.87%, respectively, at the highest concentration of 100 µg/mL. These results demonstrate that *M. pruriens* extracts decreased p-JNK and p-ERK1/2 protein expression related to inflammation in LPS-stimulated RAW 264.7 macrophages.

As a transcription factor involved in the activation, immune function, and inflammatory response of vascular endothelial cells, nuclear factor kappa-light-chain-enhancer of activated B cells (NF-κB) is present in almost all cells. In addition, NF-κB is known as a converging pathway in the expression pathway of inflammation-mediated cytokines and promotes transcription by binding to the promoter region of the target gene [10,11].

**Figure 5:**
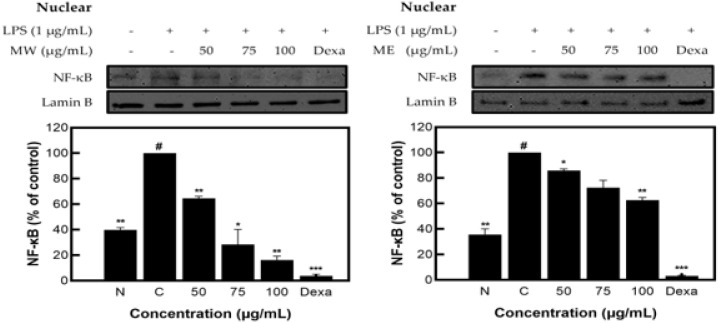
NF-κB protein expression rate of *M. pruriens* extract on macrophage cell (RAW 264.7). MW: *M. pruriens* extracted with hot water; ME: *M. pruriens* extracted with 70% ethanol; Dexa: dexamethasone. N: normal, not treated with LPS; C: control, LPS-treated. Results are means ± SD of triplicate data (significantly different according to a Tukey test and significant as compared to control. # *p* < 0.05 vs. non-treated N, * *p* < 0.05, ** *p* < 0.01, *** *p* < 0.001).

As a result of measuring the expression pattern of NF-κB protein in the nucleus of *M. pruriens* extracts, inhibitory activity of 85.42% for MW and 35.60% for ME was identified (Figure 5).

### 2.4. Identification of L-DOPA Content in Mucuna Pruriens through UPLC

As a precursor of dopamine—a neurotransmitter present in the central nervous system—L-DOPA is converted to dopamine after passing through the blood−brain barrier as a drug and is mainly used to treat Parkinson’s disease. Dopamine is also known to have the effect of improving eyesight and contrast sensitivity and reducing fixation scotoma [12].

**Figure 6:**
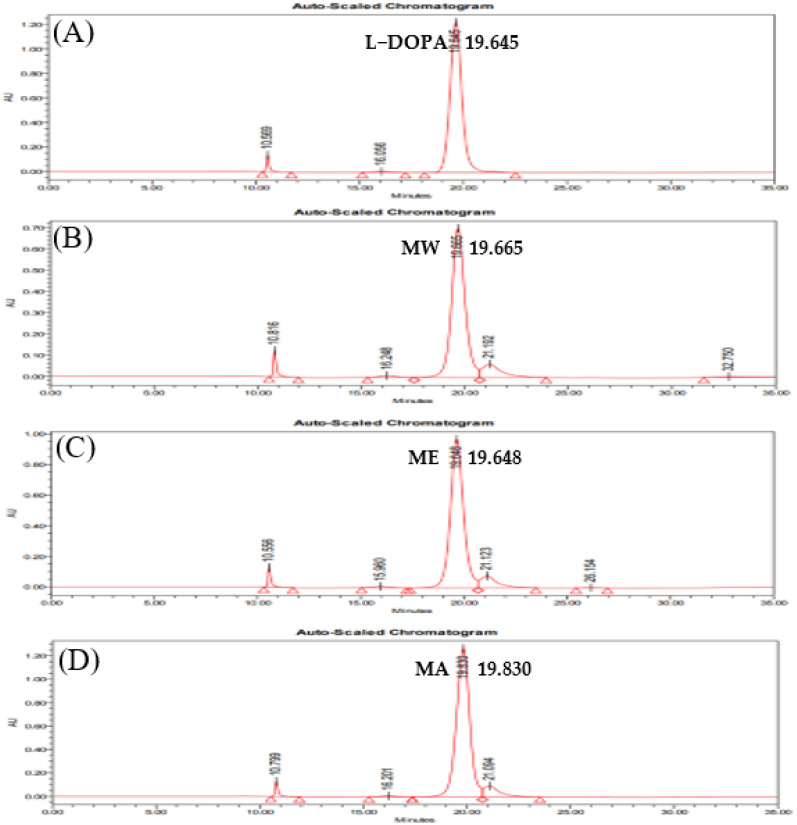
UPLC for identification of L-DOPA in *M. pruriens* extracts at 210 nm. (**A**) Standard material, (**B**) MW, (**C**) ME, and (**D**) MA.

The standard material, L-DOPA, demonstrated a peak at an RT of 19.645 min, and L-DOPA content was measured to be 252.27 mg/g at 1000 μg/mL. In contrast, peaks of MW, ME, and MA were observed at RTs of 19.665, 19.648, and 19.830 min, respectively; content was measured to be 164.62 mg/g for MW, 221.65 mg/g for ME, and 292.12 mg for MA at 4000 μg/mL (Table 1, Figure 6).

## 3. Materials and Methods

### 3.1. Preparation of Mucuna Pruriens Extract

The *M. pruriens* used in this study was purchased from Mirae Farming (Gyeonggido, Korea). To extract *M. pruriens* with hot water (MW), 100 g of crushed *M. pruriens* was placed into an Erlenmeyer flask and, after applying distilled water corresponding to approximately 10 times the specimen weight, the process of double boiling for 3 h in a 99 °C water bath was repeated in triplicate. To produce *M. pruriens* extracts with 70% ethanol (ME) and 70% acetone (MA), 100 g of crushed *M. pruriens* was placed into an Erlenmeyer flask; after immersing the equivalent to about 10 times the specimen weight in either 70% ethanol or 70% acetone, the process of extraction for 24 h at room temperature was repeated in triplicate. Then, each extract was filtered using filter paper (No. 20 filter paper; Hyundai Micro Co., Ltd., Seoul, Korea), concentrated under reduced pressure, and freeze-dried to produce powdery extract specimens of *M. pruriens*. The yields of MW, ME, and MA amounted to 15.10%, 13.02%, and 11.71%, respectively; the specimens were stored at −80 °C and used for this study [13,14].

### 3.2. Reagents

The cell line—macrophage cell (RAW 264.7)—used for the measurement of cell viability was purchased from the Korean Cell Line Bank (Seoul, Korea). The reagents for cell culture, fetal bovine serum (FBS), and Dulbecco’s modified eagle medium (DMEM) were purchased from Sigma Aldrich Co., Ltd. (St. Louis, MO, USA). The reagents used for anti-inflammatory measurement experiments, MTT, Griess reagent, RIPA lysis and extraction buffer, LPS, protease inhibitor, phosphatase inhibitor, and nuclear and cytoplasmic extraction reagents were purchased from Sigma Aldrich Co., Ltd. The iNOS antibody, COX−2 antibody, donkey anti-mouse IgG-HRP, and mouse anti-rabbit IgG-HRP for the Western blot analysis were purchased from Santa Cruz Biotechnology (Paso Robles, CA, USA). JNK antibody, p-JNK antibody, ERK1/2 antibody, p-ERK1/2 antibody, and NF-kB (p65) antibody were obtained from Cell Signaling Technology (Beverly, MA, USA).

### 3.3. Equipment Used for Experiments

The following equipment was used in the experiments in the present study: an ELISA reader (SpectraMax 190; Molecular devices, Sunnyvale, CA, USA), freeze dryer (Ilshin, Korea), pH meter (Metrohm, Switzerland), microscope (Olympus Co., Ltd., Tokyo, Japan), rotary vacuum evaporator (Rikakikai Co., Ltd., Tokyo, Japan), digital reciprocating shaker (Daihan Scientific Co., Ltd., Seoul, Korea), hot plate (Young Ji Hana Tech, Seoul, Korea), Western blot imaging system (CAS-400SM, Davinch-K Co., Ltd., Seoul, Korea), CO_2_ incubator (VS-9160GC, Hanbaek Scientific Co., Deajeon, Korea), UPLC H-Class (Waters, Waltham, MA, USA), UV detector (BAP ACQUITY UPLC TUV detector; Waters, Waltham, MA, USA), and a Hypersil GOLD ^™^ amino column (4.6 × 150 mm, 3 μm) (Thermo Fisher Scientific ^™^, Waltham, MA, USA).

### 3.4. Cell Culture

For the culturing of RAW 264.7 cells used in the present study, 10% FBS and 1% penicillin–streptomycin with added DMEM were used; it was sub-cultured after being adapted to 37 °C in a 5% CO_2_ incubator.

### 3.5. Cell Viability

The experiment to measure cytotoxicity was conducted according to the method of Carmichael et al. (1987) [15]. A total of 180 μL of RAW 264.7 cells was dispensed onto a 96-well plate at a rate of 5 × 10^3^ cells/well. After adding 20 μL each of the prepared specimens by concentration (10, 25, 50, 75, and 100 μg/mL), it was cultured at 37 °C in a 5% CO_2_ incubator for 24 h. After being cultured, 20 μL of MTT (2.5 mg/mL) solution was added and reacted for 2 h. After the reaction, the culture medium was removed, and 100 μL of dimethyl sulfoxide (DMSO) was added to each well. After reacting at room temperature for 15 min, absorbance was measured at 540 nm. The measurement of cytotoxicity was expressed as the absorbance reduction rate of the group with and without the addition of the specimen solution.

### 3.6. Nitric Oxide (NO) Inhibition Activity Assay

Measurement of NO inhibition activity was conducted using the method from Grayand et al. (1975) [16]. The amount of NO present in the supernatant of RAW 264.7 cell was measured as nitrite and nitrate, and Griess reagent was used for detection and quantification of nitrite and nitrate. After dispensing RAW 264.7 cells onto a 96-well plate at a rate of 5 × 10^4^ cells/well, it was replaced with a serum-free medium when the confluence reached 80%; then, 20 μL of specimens prepared by concentration and 1 μg/mL concentration of LPS were added to each well at a rate of 20 μL, except for the untreated group, and stimulated for 24 h. For NO production, 100 μL of supernatant and 100 μL of Griess reagent were reacted at room temperature for 20 min, and then, absorbance was measured at 540 nm.

### 3.7. Western Blot

RAW 264.7 cells were dispensed onto a 6-well cell culture plate at a rate of 5 × 10^5^ cells/well and, after replacing DMEM with a serum-free medium when confluence reached 80%, 200 μL of the samples diluted by concentration and 200 μL of LPS (1 μg/mL) were treated and cultured for 24 h. After removing the supernatant, it was washed twice with PBS and, then, the lysis buffer was added to lyse the cells, harvested with a scraper, and centrifuged (4 °C, 12,000 rpm) for 15 min. The proteins obtained by centrifugation were quantified by bicinchoninic acid (BCA) assay using bovine serum albumin (BSA) as the standard material and electrophoresed at 100 V for 2 h using 10% SDS-polyacrylamide gel. After electrophoresis was completed, transfer was conducted at 0.25 A for 1 h or more to transfer the protein to the membrane. Then, the background was removed by blocking with 5% skim milk for 2 h. Next, the primary antibody (iNOS, COX−2, JNK, p-JNK, ERK1/2, p-ERK1/2, NF-κB) (1:1000) was left overnight at 4 °C and, after washing with TBST three times or more, the secondary antibody (donkey anti-mouse IgG-HRP, mouse anti-rabbit IgG-HRP) (1:1000) was reacted for 1.5 h. After the reaction, washing with Tris-buffered saline−Tween 20 (TBST) was repeated three times or more and, then, protein expression was measured using a Western blot imaging system [17].

### 3.8. Ultra-Performance Liquid Chromatography (UPLC)

L-DOPA (Sigma-Aldrich, St. Louis, MO, USA) was analyzed with a UPLC system consisting of UPLC H-Class (BAP ACQUITY UPLC TUV detector; Waters, Waltham, MA, USA), and its separation was conducted using a Hypersil GOLD^™^ Amino column (4.6 × 150 mm, 3 μm; Thermo Fisher Scientific ^™^, Waltham, MA, USA). (A) 0.1% phosphoric acid in deionized water and (B) acetonitrile were used as mobile phase solvents. L-DOPA analysis was conducted under the elution conditions of (A) 0−5 min, 95%; (A) 5−35 min, 95−0%; (B) 35–45 min, 100%; and (A) 45−55 min, 95%. The flow rate was 0.1 μL/min, and detection was performed at 210 nm. L-DOPA analysis of MW, ME, and MA was identified by comparing the retention time of L-DOPA, a standard material, under the same analysis conditions [18,19].

### 3.9. Statistical Analysis

Statistical analysis of all experimental results was expressed by mean and standard deviation using IBM SPSS Statistics (version 20.0, IBM Corp., Armonk, NY, USA) and analysis of variance (ANOVA). After confirming significance, multiple comparisons using Duncan’s multiple range tests were performed and analyzed at the significance level *p* < 0.05.

## 4. Conclusions

In summary, after analyzing the cytotoxicity of MW, ME, and MA to macrophage cells (RAW 264.7) using the MTT assay, a concentration of 100 μg/mL, which prevents the possibility of cell population degradation, was applied to the cell experiment. As a result of performing the nitric oxide inhibition activity assay, MW, ME, and MA showed NO inhibition activity; among them, MW and ME demonstrated the best activity and were selected for Western blot analysis. After identifying the expression patterns of inflammation-related proteins such as iNOS, COX−2, JNK, ERK1/2, and NF-κB through Western blot analysis, both MW and ME showed inhibition patterns. Following the analysis of L-DOPA contained in *M. pruriens* through UPLC, high L-DOPA content was detected in MW, ME, and MA. Based on the results of the experiment, it can be concluded that *M. pruriens* extracts might have beneficial anti-inflammatory uses.

## Figures and Tables

**Table 1:** L-DOPA content in *M. pruriens* extracts.

Sample	Peak Results				
	RT (min)	Peak Area	Peak Height	Peak Area (%)	Content (mg/g)
MW (4,000 µg/mL)	19.665	31,961,556	703,171	81.92	164.62
ME (4,000 µg/mL)	19.648	43,366,295	971,505	87.45	221.65
MA (4,000 µg/mL)	19.830	57,459,871	1267,687	89.49	292.12
L-DOPA	19.645	49,491,205	1224,580	96.44	252.27

MW: *M. pruriens* extracted with hot water; ME: *M. pruriens* extracted with 70% ethanol; MA: *M. pruriens* extracted with 70% acetone; RT: retention time.

## Data Availability

Not applicable.
